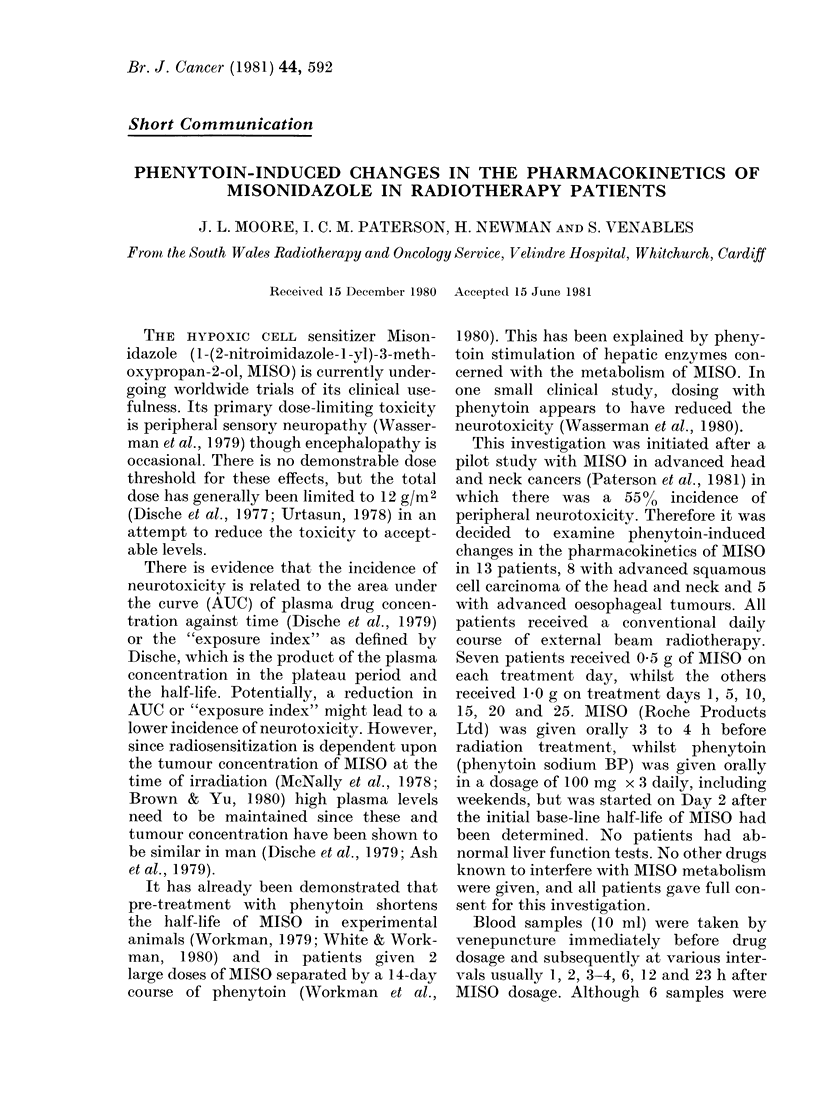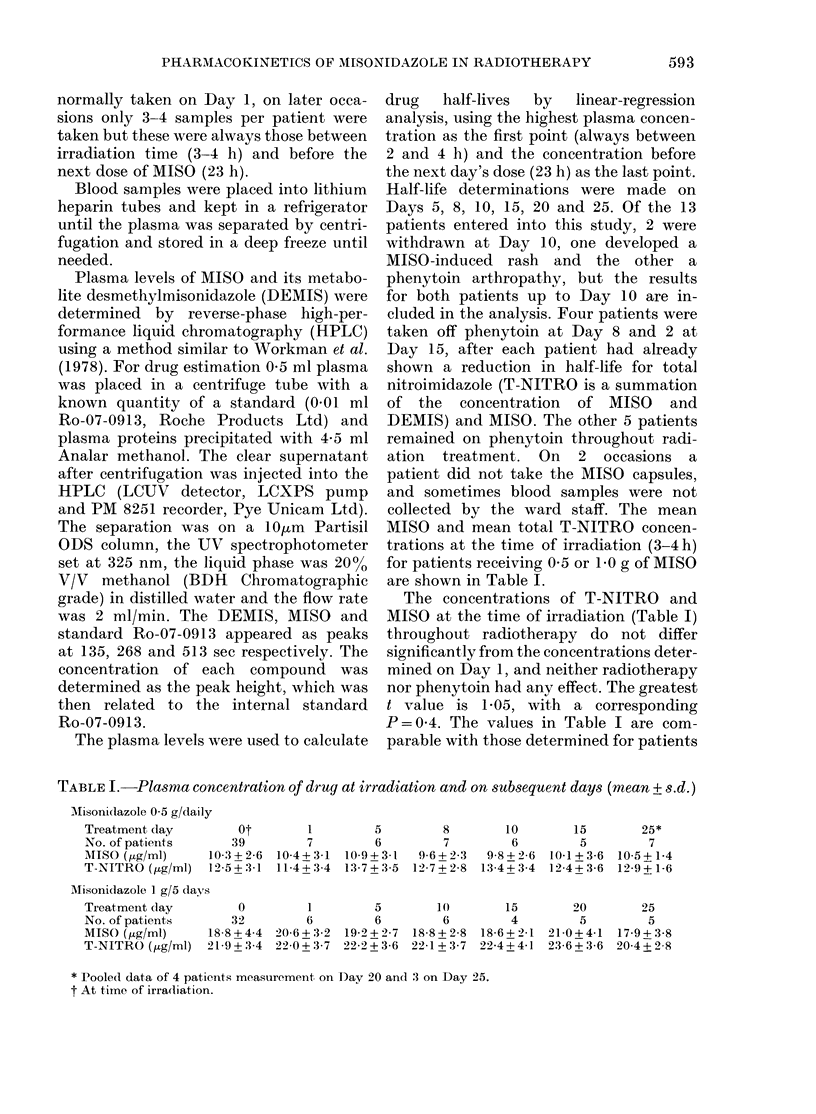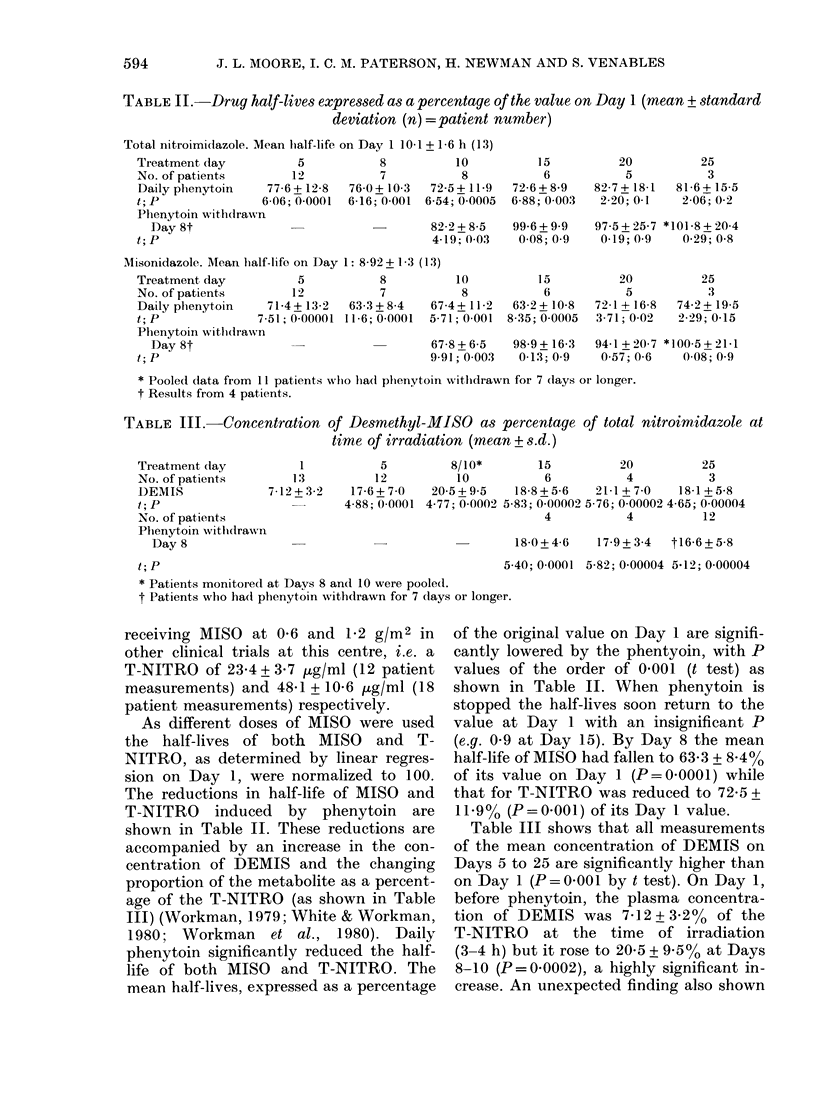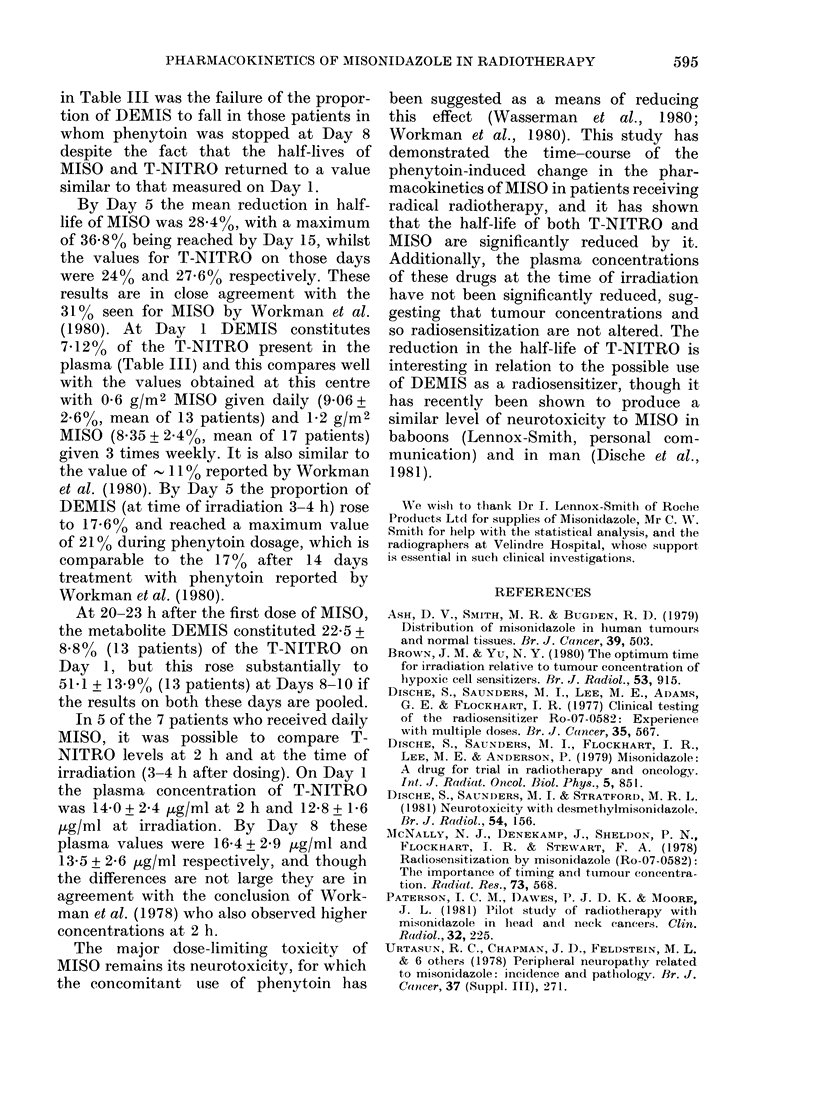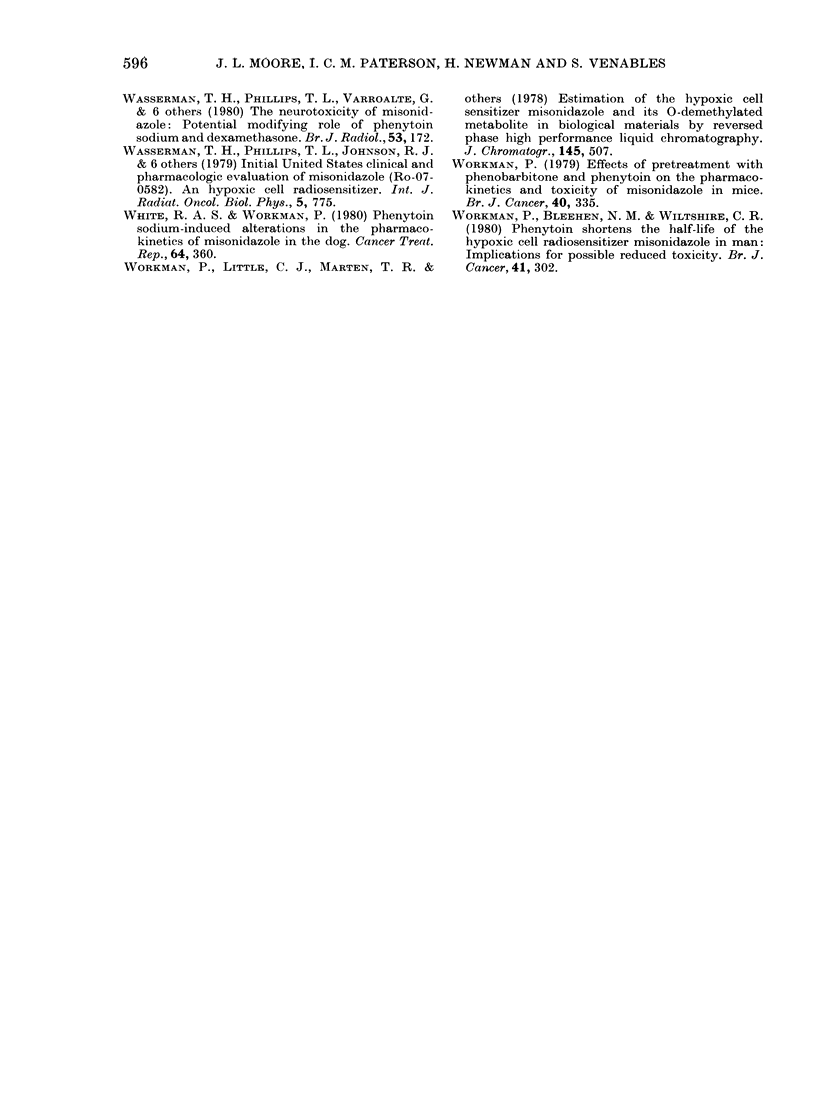# Phenytoin-induced changes in the pharmacokinetics of misonidazole in radiotherapy patients.

**DOI:** 10.1038/bjc.1981.232

**Published:** 1981-10

**Authors:** J. L. Moore, I. C. Paterson, H. Newman, S. Venables


*Br. J. Cancer* (1981) **44**, 592

***Short Communication***

### PHENYTOIN-INDUCED CHANGES IN THE PHARMACOKINETICS OF MISONIDAZOLE IN RADIOTHERAPY PATIENTS

J. L. MOORE, I. C. M. PATERSON, H. NEWMAN AND S. VENABLES

*From the South Wales Radiotherapy and Oncology Service, Velindre Hospital, Whitchurch, Cardiff*

Received 15 December 1980 Accepted 15 June 1981

THE HYPOXIC CELL sensitizer Misonidazole (1-(2-nitroimidazole-1-yl)-3-methoxypropan-2-ol, MISO) is currently undergoing worldwide trials of its clinical usefulness. Its primary dose-limiting toxicity is peripheral sensory neuropathy (Wasserman *et al.*, 1979) though encephalopathy is occasional. There is no demonstrable dose threshold for these effects, but the total dose has generally been limited to 12 g/m$^2$ (Dische *et al.*, 1977; Urtasun, 1978) in an attempt to reduce the toxicity to acceptable levels.

There is evidence that the incidence of neurotoxicity is related to the area under the curve (AUC) of plasma drug concentration against time (Dische *et al.*, 1979) or the "exposure index" as defined by Dische, which is the product of the plasma concentration in the plateau period and the half-life. Potentially, a reduction in AUC or "exposure index" might lead to a lower incidence of neurotoxicity. However, since radiosensitization is dependent upon the tumour concentration of MISO at the time of irradiation (McNally *et al.*, 1978; Brown & Yu, 1980) high plasma levels need to be maintained since these and tumour concentration have been shown to be similar in man (Dische *et al.*, 1979; Ash *et al.*, 1979).

It has already been demonstrated that pre-treatment with phenytoin shortens the half-life of MISO in experimental animals (Workman, 1979; White & Workman, 1980) and in patients given 2 large doses of MISO separated by a 14-day course of phenytoin (Workman *et al.*, 1980). This has been explained by phenytoin stimulation of hepatic enzymes concerned with the metabolism of MISO. In one small clinical study, dosing with phenytoin appears to have reduced the neurotoxicity (Wasserman *et al.*, 1980).

This investigation was initiated after a pilot study with MISO in advanced head and neck cancers (Paterson *et al.*, 1981) in which there was a 55% incidence of peripheral neurotoxicity. Therefore it was decided to examine phenytoin-induced changes in the pharmacokinetics of MISO in 13 patients, 8 with advanced squamous cell carcinoma of the head and neck and 5 with advanced oesophageal tumours. All patients received a conventional daily course of external beam radiotherapy. Seven patients received 0·5 g of MISO on each treatment day, whilst the others received 1·0 g on treatment days 1, 5, 10, 15, 20 and 25. MISO (Roche Products Ltd) was given orally 3 to 4 h before radiation treatment, whilst phenytoin (phenytoin sodium BP) was given orally in a dosage of 100 mg × 3 daily, including weekends, but was started on Day 2 after the initial base-line half-life of MISO had been determined. No patients had abnormal liver function tests. No other drugs known to interfere with MISO metabolism were given, and all patients gave full consent for this investigation.

Blood samples (10 ml) were taken by venepuncture immediately before drug dosage and subsequently at various intervals usually 1, 2, 3–4, 6, 12 and 23 h after MISO dosage. Although 6 samples were normally taken on Day 1, on later occasions only 3–4 samples per patient were taken but these were always those between irradiation time (3–4 h) and before the next dose of MISO (23 h).

Blood samples were placed into lithium heparin tubes and kept in a refrigerator until the plasma was separated by centrifugation and stored in a deep freeze until needed.

Plasma levels of MISO and its metabolite desmethylmisonidazole (DEMIS) were determined by reverse-phase high-performance liquid chromatography (HPLC) using a method similar to Workman *et al.* (1978). For drug estimation 0·5 ml plasma was placed in a centrifuge tube with a known quantity of a standard (0·01 ml Ro-07-0913, Roche Products Ltd) and plasma proteins precipitated with 4·5 ml Analar methanol. The clear supernatant after centrifugation was injected into the HPLC (LCUV detector, LCXPS pump and PM 8251 recorder, Pye Unicam Ltd). The separation was on a 10$\mu$m Partisil ODS column, the UV spectrophotometer set at 325 nm, the liquid phase was 20% V/V methanol (BDH Chromatographic grade) in distilled water and the flow rate was 2 ml/min. The DEMIS, MISO and standard Ro-07-0913 appeared as peaks at 135, 268 and 513 sec respectively. The concentration of each compound was determined as the peak height, which was then related to the internal standard Ro-07-0913.

The plasma levels were used to calculate drug half-lives by linear-regression analysis, using the highest plasma concentration as the first point (always between 2 and 4 h) and the concentration before the next day's dose (23 h) as the last point. Half-life determinations were made on Days 5, 8, 10, 15, 20 and 25. Of the 13 patients entered into this study, 2 were withdrawn at Day 10, one developed a MISO-induced rash and the other a phenytoin arthropathy, but the results for both patients up to Day 10 are included in the analysis. Four patients were taken off phenytoin at Day 8 and 2 at Day 15, after each patient had already shown a reduction in half-life for total nitroimidazole (T-NITRO is a summation of the concentration of MISO and DEMIS) and MISO. The other 5 patients remained on phenytoin throughout radiation treatment. On 2 occasions a patient did not take the MISO capsules, and sometimes blood samples were not collected by the ward staff. The mean MISO and mean total T-NITRO concentrations at the time of irradiation (3–4 h) for patients receiving 0·5 or 1·0 g of MISO are shown in Table I.

The concentrations of T-NITRO and MISO at the time of irradiation (Table I) throughout radiotherapy do not differ significantly from the concentrations determined on Day 1, and neither radiotherapy nor phenytoin had any effect. The greatest $t$ value is 1·05, with a corresponding $P = 0{\cdot}4$. The values in Table I are comparable with those determined for patients

TABLE I.—*Plasma concentration of drug at irradiation and on subsequent days* (*mean* ± *s.d.*)

Misonidazole 0·5 g/daily

| Treatment day | 0† | 1 | 5 | 8 | 10 | 15 | 25* |
|---|---|---|---|---|---|---|---|
| No. of patients | 39 | 7 | 6 | 7 | 6 | 5 | 7 |
| MISO ($\mu$g/ml) | 10·3 ± 2·6 | 10·4 ± 3·1 | 10·9 ± 3·1 | 9·6 ± 2·3 | 9·8 ± 2·6 | 10·1 ± 3·6 | 10·5 ± 1·4 |
| T-NITRO ($\mu$g/ml) | 12·5 ± 3·1 | 11·4 ± 3·4 | 13·7 ± 3·5 | 12·7 ± 2·8 | 13·4 ± 3·4 | 12·4 ± 3·6 | 12·9 ± 1·6 |

Misonidazole 1 g/5 days

| Treatment day | 0 | 1 | 5 | 10 | 15 | 20 | 25 |
|---|---|---|---|---|---|---|---|
| No. of patients | 32 | 6 | 6 | 6 | 4 | 5 | 5 |
| MISO ($\mu$g/ml) | 18·8 ± 4·4 | 20·6 ± 3·2 | 19·2 ± 2·7 | 18·8 ± 2·8 | 18·6 ± 2·1 | 21·0 ± 4·1 | 17·9 ± 3·8 |
| T-NITRO ($\mu$g/ml) | 21·9 ± 3·4 | 22·0 ± 3·7 | 22·2 ± 3·6 | 22·1 ± 3·7 | 22·4 ± 4·1 | 23·6 ± 3·6 | 20·4 ± 2·8 |

* Pooled data of 4 patients measurement on Day 20 and 3 on Day 25.
† At time of irradiation.

TABLE II.—*Drug half-lives expressed as a percentage of the value on Day* 1 (*mean* ± *standard deviation* (*n*) = *patient number*)

Total nitroimidazole. Mean half-life on Day 1 10·1 ± 1·6 h (13)

| Treatment day | 5 | 8 | 10 | 15 | 20 | 25 |
|---|---|---|---|---|---|---|
| No. of patients | 12 | 7 | 8 | 6 | 5 | 3 |
| Daily phenytoin | 77·6 ± 12·8 | 76·0 ± 10·3 | 72·5 ± 11·9 | 72·6 ± 8·9 | 82·7 ± 18·1 | 81·6 ± 15·5 |
| *t*; *P* | 6·06; 0·0001 | 6·16; 0·001 | 6·54; 0·0005 | 6·88; 0·003 | 2·20; 0·1 | 2·06; 0·2 |
| Phenytoin withdrawn Day 8† | — | — | 82·2 ± 8·5 | 99·6 ± 9·9 | 97·5 ± 25·7 | *101·8 ± 20·4 |
| *t*; *P* | | | 4·19; 0·03 | 0·08; 0·9 | 0·19; 0·9 | 0·29; 0·8 |

Misonidazole. Mean half-life on Day 1: 8·92 ± 1·3 (13)

| Treatment day | 5 | 8 | 10 | 15 | 20 | 25 |
|---|---|---|---|---|---|---|
| No. of patients | 12 | 7 | 8 | 6 | 5 | 3 |
| Daily phenytoin | 71·4 ± 13·2 | 63·3 ± 8·4 | 67·4 ± 11·2 | 63·2 ± 10·8 | 72·1 ± 16·8 | 74·2 ± 19·5 |
| *t*; *P* | 7·51; 0·00001 | 11·6; 0·0001 | 5·71; 0·001 | 8·35; 0·0005 | 3·71; 0·02 | 2·29; 0·15 |
| Phenytoin withdrawn Day 8† | — | — | 67·8 ± 6·5 | 98·9 ± 16·3 | 94·1 ± 20·7 | *100·5 ± 21·1 |
| *t*; *P* | | | 9·91; 0·003 | 0·13; 0·9 | 0·57; 0·6 | 0·08; 0·9 |

* Pooled data from 11 patients who had phenytoin withdrawn for 7 days or longer.
† Results from 4 patients.

TABLE III.—*Concentration of Desmethyl-MISO as percentage of total nitroimidazole at time of irradiation* (*mean* ± *s.d.*)

| Treatment day | 1 | 5 | 8/10* | 15 | 20 | 25 |
|---|---|---|---|---|---|---|
| No. of patients | 13 | 12 | 10 | 6 | 4 | 3 |
| DEMIS | 7·12 ± 3·2 | 17·6 ± 7·0 | 20·5 ± 9·5 | 18·8 ± 5·6 | 21·1 ± 7·0 | 18·1 ± 5·8 |
| *t*; *P* | — | 4·88; 0·0001 | 4·77; 0·0002 | 5·83; 0·00002 | 5·76; 0·00002 | 4·65; 0·00004 |
| No. of patients | | | | 4 | 4 | 12 |
| Phenytoin withdrawn Day 8 | — | — | — | 18·0 ± 4·6 | 17·9 ± 3·4 | †16·6 ± 5·8 |
| *t*; *P* | | | | 5·40; 0·0001 | 5·82; 0·00004 | 5·12; 0·00004 |

* Patients monitored at Days 8 and 10 were pooled.
† Patients who had phenytoin withdrawn for 7 days or longer.

receiving MISO at 0·6 and 1·2 g/m$^2$ in other clinical trials at this centre, *i.e.* a T-NITRO of 23·4 ± 3·7 μg/ml (12 patient measurements) and 48·1 ± 10·6 μg/ml (18 patient measurements) respectively.

As different doses of MISO were used the half-lives of both MISO and T-NITRO, as determined by linear regression on Day 1, were normalized to 100. The reductions in half-life of MISO and T-NITRO induced by phenytoin are shown in Table II. These reductions are accompanied by an increase in the concentration of DEMIS and the changing proportion of the metabolite as a percentage of the T-NITRO (as shown in Table III) (Workman, 1979; White & Workman, 1980; Workman *et al.*, 1980). Daily phenytoin significantly reduced the half-life of both MISO and T-NITRO. The mean half-lives, expressed as a percentage of the original value on Day 1 are significantly lowered by the phentyoin, with *P* values of the order of 0·001 (*t* test) as shown in Table II. When phenytoin is stopped the half-lives soon return to the value at Day 1 with an insignificant *P* (*e.g.* 0·9 at Day 15). By Day 8 the mean half-life of MISO had fallen to 63·3 ± 8·4% of its value on Day 1 ($P = 0·0001$) while that for T-NITRO was reduced to 72·5 ± 11·9% ($P = 0·001$) of its Day 1 value.

Table III shows that all measurements of the mean concentration of DEMIS on Days 5 to 25 are significantly higher than on Day 1 ($P = 0·001$ by *t* test). On Day 1, before phenytoin, the plasma concentration of DEMIS was 7·12 ± 3·2% of the T-NITRO at the time of irradiation (3–4 h) but it rose to 20·5 ± 9·5% at Days 8–10 ($P = 0·0002$), a highly significant increase. An unexpected finding also shown in Table III was the failure of the proportion of DEMIS to fall in those patients in whom phenytoin was stopped at Day 8 despite the fact that the half-lives of MISO and T-NITRO returned to a value similar to that measured on Day 1.

By Day 5 the mean reduction in half-life of MISO was 28·4%, with a maximum of 36·8% being reached by Day 15, whilst the values for T-NITRO on those days were 24% and 27·6% respectively. These results are in close agreement with the 31% seen for MISO by Workman *et al.* (1980). At Day 1 DEMIS constitutes 7·12% of the T-NITRO present in the plasma (Table III) and this compares well with the values obtained at this centre with 0·6 g/m$^2$ MISO given daily (9·06 ± 2·6%, mean of 13 patients) and 1·2 g/m$^2$ MISO (8·35 ± 2·4%, mean of 17 patients) given 3 times weekly. It is also similar to the value of ~11% reported by Workman *et al.* (1980). By Day 5 the proportion of DEMIS (at time of irradiation 3–4 h) rose to 17·6% and reached a maximum value of 21% during phenytoin dosage, which is comparable to the 17% after 14 days treatment with phenytoin reported by Workman *et al.* (1980).

At 20–23 h after the first dose of MISO, the metabolite DEMIS constituted 22·5 ± 8·8% (13 patients) of the T-NITRO on Day 1, but this rose substantially to 51·1 ± 13·9% (13 patients) at Days 8–10 if the results on both these days are pooled.

In 5 of the 7 patients who received daily MISO, it was possible to compare T-NITRO levels at 2 h and at the time of irradiation (3–4 h after dosing). On Day 1 the plasma concentration of T-NITRO was 14·0 ± 2·4 μg/ml at 2 h and 12·8 ± 1·6 μg/ml at irradiation. By Day 8 these plasma values were 16·4 ± 2·9 μg/ml and 13·5 ± 2·6 μg/ml respectively, and though the differences are not large they are in agreement with the conclusion of Workman *et al.* (1978) who also observed higher concentrations at 2 h.

The major dose-limiting toxicity of MISO remains its neurotoxicity, for which the concomitant use of phenytoin has been suggested as a means of reducing this effect (Wasserman *et al.*, 1980; Workman *et al.*, 1980). This study has demonstrated the time–course of the phenytoin-induced change in the pharmacokinetics of MISO in patients receiving radical radiotherapy, and it has shown that the half-life of both T-NITRO and MISO are significantly reduced by it. Additionally, the plasma concentrations of these drugs at the time of irradiation have not been significantly reduced, suggesting that tumour concentrations and so radiosensitization are not altered. The reduction in the half-life of T-NITRO is interesting in relation to the possible use of DEMIS as a radiosensitizer, though it has recently been shown to produce a similar level of neurotoxicity to MISO in baboons (Lennox-Smith, personal communication) and in man (Dische *et al.*, 1981).

We wish to thank Dr I. Lennox-Smith of Roche Products Ltd for supplies of Misonidazole, Mr C. W. Smith for help with the statistical analysis, and the radiographers at Velindre Hospital, whose support is essential in such clinical investigations.

### REFERENCES

ASH, D. V., SMITH, M. R. & BUGDEN, R. D. (1979) Distribution of misonidazole in human tumours and normal tissues. *Br. J. Cancer*, **39**, 503.

BROWN, J. M. & YU, N. Y. (1980) The optimum time for irradiation relative to tumour concentration of hypoxic cell sensitizers. *Br. J. Radiol.*, **53**, 915.

DISCHE, S., SAUNDERS, M. I., LEE, M. E., ADAMS, G. E. & FLOCKHART, I. R. (1977) Clinical testing of the radiosensitizer Ro-07-0582: Experience with multiple doses. *Br. J. Cancer*, **35**, 567.

DISCHE, S., SAUNDERS, M. I., FLOCKHART, I. R., LEE, M. E. & ANDERSON, P. (1979) Misonidazole: A drug for trial in radiotherapy and oncology. *Int. J. Radiat. Oncol. Biol. Phys.*, **5**, 851.

DISCHE, S., SAUNDERS, M. I. & STRATFORD, M. R. L. (1981) Neurotoxicity with desmethylmisonidazole. *Br. J. Radiol.*, **54**, 156.

MCNALLY, N. J., DENEKAMP, J., SHELDON, P. N., FLOCKHART, I. R. & STEWART, F. A. (1978) Radiosensitization by misonidazole (Ro-07-0582): The importance of timing and tumour concentration. *Radiat. Res.*, **73**, 568.

PATERSON, I. C. M., DAWES, P. J. D. K. & MOORE, J. L. (1981) Pilot study of radiotherapy with misonidazole in head and neck cancers. *Clin. Radiol.*, **32**, 225.

URTASUN, R. C., CHAPMAN, J. D., FELDSTEIN, M. L. & 6 others (1978) Peripheral neuropathy related to misonidazole: incidence and pathology. *Br. J. Cancer*, **37** (Suppl. III), 271.

WASSERMAN, T. H., PHILLIPS, T. L., VARROALTE, G. & 6 others (1980) The neurotoxicity of misonidazole: Potential modifying role of phenytoin sodium and dexamethasone. *Br. J. Radiol.*, **53**, 172.

WASSERMAN, T. H., PHILLIPS, T. L., JOHNSON, R. J. & 6 others (1979) Initial United States clinical and pharmacologic evaluation of misonidazole (Ro-07-0582). An hypoxic cell radiosensitizer. *Int. J. Radiat. Oncol. Biol. Phys.*, **5**, 775.

WHITE, R. A. S. & WORKMAN, P. (1980) Phenytoin sodium-induced alterations in the pharmacokinetics of misonidazole in the dog. *Cancer Treat. Rep.*, **64**, 360.

WORKMAN, P., LITTLE, C. J., MARTEN, T. R. & others (1978) Estimation of the hypoxic cell sensitizer misonidazole and its O-demethylated metabolite in biological materials by reversed phase high performance liquid chromatography. *J. Chromatogr.*, **145**, 507.

WORKMAN, P. (1979) Effects of pretreatment with phenobarbitone and phenytoin on the pharmacokinetics and toxicity of misonidazole in mice. *Br. J. Cancer*, **40**, 335.

WORKMAN, P., BLEEHEN, N. M. & WILTSHIRE, C. R. (1980) Phenytoin shortens the half-life of the hypoxic cell radiosensitizer misonidazole in man: Implications for possible reduced toxicity. *Br. J. Cancer*, **41**, 302.